# Prelacteal feeding practices in Pakistan: a mixed-methods study

**DOI:** 10.1186/s13006-020-00295-8

**Published:** 2020-06-08

**Authors:** Muhammad Asim, Zarak Husain Ahmed, Mark D. Hayward, Elizabeth M. Widen

**Affiliations:** 1 Department of Community Health Sciences, Aga Khan University, Karachi, Pakistan; 2 Department of Sociology, University of Sargodha, Sargodha, Pakistan; 3 Population Research Center, University of Texas at Austin, Austin, USA; 4 Department of Sociology & Population Research Center, University of Texas at Austin, Austin, USA; 5 Department of Nutritional Sciences & Population Research Center, University of Texas at Austin, Austin, Texas USA

**Keywords:** Prelacteal, Delayed breastfeeding, Insufficient breast milk, Home and hospital deliveries, Pakistan

## Abstract

**Background:**

Prelacteal feeding, the feeding a newborn substances or liquids before breastfeeding, is a common cultural practice in Pakistan, but is associated with neonatal morbidity and mortality because it delays early initiation of breastfeeding. In this study, we sought to examine the social and cultural factors associated with prelacteal feeding in Pakistan.

**Methods:**

This mixed-method study used data from the Pakistan Demographic and Health Survey (PDHS) 2012–13. Findings from the survey were complemented by qualitative interviews with mothers and healthcare providers. In a subset of PDHS dyads (*n* = 1361) with children (0–23 months), descriptive statistics and bivariate and multivariable logistic regression analysis examined factors associated with prelacteal feeding. The qualitative study included in-depth interviews with six mothers and six health care providers, which were analyzed using NVivo software version 10.

**Results:**

In PDHS, a majority of children (64.7%) received prelacteal feeding. The most common prelacteal food was milk other than breast milk (24.5%), while over a fifth (21.8%) of mothers reported giving honey and sugar water. Factors associated with prelacteal feeding included: birth at public health facilities (AOR 0.46, 95% CI 0.02, 0.95), maternal primary education (AOR 2.28, 95% CI 1.35, 3.85), and delayed breastfeeding initiation (AOR 0.03, 95% CI 0.01, 0.61). In our qualitative study, the major themes found associated with prelacteal feedings included: easy access to prelacteal substances at health facilities, deliveries in private health facilities, prelacteals as a family tradition for socialization, insufficient breast milk, Sunna of Holy Prophet, and myths about colostrum.

**Conclusions:**

These data indicate that prelacteal feeding is a well-established practice and social norm in Pakistan. Policies and interventions aimed at promoting breastfeeding need to take these customs into consideration to achieve the desired behavioral changes.

## Background

Nutrition and care in the neonatal period are critical for infant survival, growth, and development. The World Health Organization (WHO) recommends initiation of breastfeeding within the first hour of birth and exclusive breastfeeding for the first 6 months of life, meaning no other foods or liquids should be introduced to the infant during that time [1]. However, the practice of feeding substances or liquids other than breast milk to newborns is a common cultural practice in many low-income countries [2]. Providing different substances to infants before the initiation of breastfeeding, or in the first 3 days after delivery, is known as prelacteal feeding [3–5], and the substances are known as prelacteals [6]. The type of prelacteal fed varies according to cultural preferences and include a diverse array of substances such as honey, goat milk, and rose or sap water [7, 8].

Prelacteal feeding is a major cause of delayed breastfeeding, non-exclusive breastfeeding, and lactation failure [9–11]. Prelacteal feeding deprives neonates of the potential benefits of colostrum, which may be a major contributor to high morbidity and mortality in the neonatal period [12–16]. This practice may lead to the development of a weak immune system and infection in children. To avoid these negative health outcomes, some believe that shifting prelacteal feeding practices could potentially save the lives of around 830,000 children annually [17].

According to data from low and middle-income countries (LMICs), the prevalence of prelacteal feeding in Ethiopia [18], India [19], Bangladesh [20], Afghanistan [21], and Nigeria [22] is 19, 21, 27, 43, and 59%, respectively. When compared to these countries, the situation is markedly worse in Pakistan, where the practice of giving prelacteal feeds to neonates increased from 68% of all births in 2007 to 76% in 2018 [23]. Consequently, Pakistan has the lowest prevalence of early initiation of breastfeeding and highest rate of non-exclusive breastfeeding in South Asia [24]. This can be attributed to culture-specific prelacteal practices, where early initiation of breastfeeding is deliberately delayed to adhere to cultural and social norms [25]. Thus, breastfeeding is, unfortunately, not an immediate priority for nursing mothers in Pakistan, many of whom believe that the first feed of neonates should be honey, rosewater, or goat milk [26, 27]. Such high prevalence of prelacteal feeding, may therefore place Pakistani neonates at a greater risk for morbidities and mortality associated with prelacteals and delayed breastfeeding than other LMICs.

To advance the understanding of prelacteal feeding practices in Pakistan, we applied a mixed-methods approach to explore the major determinants for prelacteal feeding in Pakistan. This study includes quantitative analysis with Pakistan Demographic and Health Survey (PDHS) data, as well as a qualitative assessment of healthcare providers and mothers. This approach allowed us to comprehensively examine prelacteal feeding practices in Pakistan.

## Methods

We applied a sequential exploratory mixed-methods design, which consisted of two phases: qualitative investigation, followed by quantitative data analysis and data triangulation [28]. During the first phase, we conducted in-depth interviews with mothers and healthcare providers. Subsequently, we analyzed the PDHS 2012–2013 data regarding neonatal feeding practices. Following this, we triangulated the findings of both data sets to generate a holistic picture of the sociocultural factors that influence prelacteal feeding in the Pakistani population.

### Qualitative assessment of prelacteal feeding

For in-depth understanding of sociocultural context of prelacteal feeding, semi-structured in-depth interviews were conducted with mothers and healthcare providers.

### Study participants and sampling

To help narrow down our sampling pool for the qualitative assessment, we looked to the PDHS 2012–2013 data set. According to PDHS, a majority of caretakers (87%) in the Punjab province of Pakistan administered prelacteals, and the prevalence of prelacteal feeding was much higher in Punjab compared to other provinces. Therefore, we conducted our qualitative interviews in select districts of the Punjab Pakistan’s most populous province. Three districts (Okara, Sahiwal and Pakpatan) were randomly selected out of the thirty-six districts that comprise Punjab. These three districts have a predominantly rural populations and are situated in east-central Pakistan. Informants and key-informants were selected via non-probability sampling in each district. Mothers (i.e., informants) were selected purposively based on their educational status and parity. First, we selected mothers with varying education levels (i.e., illiterate, primary, middle, and secondary). To acquire detailed information about prelacteal feeding practices, we selected mothers who had at least two children, with the youngest child aged 0–23 months. Selection criteria for healthcare providers (i.e., key-informants) included working in public or private health facility, and having more than 5 years of work experience within the community. Key-informants included health workers, midwives, nurses, and physicians. We conducted a total of twelve semi-structured in-depth interviews with informants (*n* = 6) and key informants (*n* = 6).

### Data collection

For informants, an interview guide with several probing options was designed to explore sociocultural preferences and reasons for prelacteal feeding. Similarly, a separate interview guide was used for key-informants to explore their perceptions on prelacteal feeds. Each interview was conducted at a house or health facility, and the duration of interviews ranged from 20 to 25 min. Interviews were recorded using a digital recorder and hand-written field notes were collected in the local language by a native speaker (MA). The research team collected data between March 2017 and May 2017. The first author (MA) collected data with the help of two research assistants trained in qualitative methods and with educational backgrounds in the social sciences. After reaching theme saturation during the interviews, data collection was concluded.

### Ethical consideration

Study protocols were approved by the ethical review committee of The University of Sargodha, Pakistan [UOS/Acad/399]. Additionally, the research team obtained official permission to conduct interviews with informants and key-informants from the District Health Authority in each district. All participants received a structured letter outlining study aims and procedures, and informed participants of their right to withdraw from the study at any moment and stated a promise of anonymity. Study objectives were thoroughly discussed with each participant, and written informed consent was obtained before starting the interview.

### Data analysis

Interviews were conducted in the local language (Punjabi), then transcribed verbatim and translated into English by the first author. Later, the English transcriptions were counter-checked by another co-author (ZHA) using the hand-written field notes to ensure the quality of the data. Then, the English transcriptions were uploaded into NVivo software (version 10) for data management and analysis. The inductive method was used to formulate major themes and categories from the transcripts [29]. In this approach, an exhaustive list of codes was organized through transcripts rather than utilizing a predetermined codebook. Codes were subsequently grouped under categories and themes, and a thematic matrix was developed to display coded text data. Coding discrepancies were discussed and resolved to reduce bias. To ensure the authenticity of the findings, data were triangulated by the data sources (i.e., informants, key-informants, and field observation) and reported in this study. Finally, overarching themes were discussed by the co-authors and repeated codes were reconciled during interpretation.

### Quantitative assessment of prelacteal feeding

We analyzed the PDHS data for years 2012–2013, which was the third national survey funded by the US Agency for International Development [26]. The PDHS is a nationally representative dataset on sociodemographic and mother-child health related indicators collected with representative proportions across four provinces (i.e., Punjab, Sindh, Khyber Pakhtunkhawa, and Baluchistan), along with Gilgit-Baltistan and Islamabad. We also selected the PDHS dataset because it collected information on the types of liquids or substances provided to the neonate.

While PDHS survey data are available for children ages 0–59 months, we analyzed a subset of data of mothers with children ages 0–23 months (*n* = 1361) to ensure that mothers could more accurately recall their prelacteal practices. The prelacteal feeding variable (categorical dependent, yes/no) was determined by pooling results from a battery of questions that recalled history of giving anything to the infant by mouth in the first 3 days after delivery. Relevant predictors of prelacteal feeding in PDHS were selected by conducting a review of the literature [30–34], and included sociodemographic characteristics such as: wealth quintile, region, mothers’ educational level, sex of the head of household, and type of residence. Other characteristics included: maternal age, place of delivery (categorical), type of delivery (categorical), initiation of breastfeeding (categorical), and sex of the child (categorical). Some variables were re-coded for analyses, including mother’s age (categorical), antenatal health utilization, and initiation of first breastfeed (after birth in hours and days).

Data were coded and analyzed using SPSS (version 21). Descriptive analysis, including frequency distributions and percentages, were used to examine the dependent and independent variables. Binary logistic regression analysis examined associations between sociodemographic characteristics and other independent variables with the likelihood of prelacteal feeding. Furthermore, multivariable logistic regression analysis with backward stepwise selection was performed using variables that predicted prelacteal feeding (*p* <  0.20) in binary analysis. Multicollinearity was assessed between highly correlated variables before developing the multivariate model. All independent variables had a variance inflation factor of less than 1.5.

## Results

### Qualitative results

Through in-depth semi-structured interviews, informants and key-informants were asked to identify the factors associated with prelacteal feeding. Informants were mothers and key-informants were health workers, midwives, nurses, and physicians (See Table 1). A number of sociocultural and administrative motivations were identified for feeding prelacteals to neonates. These included: delivery in a private health facility, inadequate antenatal counseling, myths about colostrum, Sunna of the Holy Prophet, family rituals, and perceptions of insufficient breast milk.

**Table 1:** Background characteristics of the informants (*n* = 6) and key-informants recruited for qualitative interviews (*n* = 6)

Codes	Informants/ key-informant	Education/ Type of employment	Age in years	Professional experience	Urbanicity of Position/Locale
1	Informant	5th grade	26	–	Rural, Sahiwal
2	Informant	Illiterate	40	–	Rural, Sahiwal
3	Informant	8th grade	30	–	Urban, Okara
4	Informant	10th grade	22	–	Rural, Okara
5	Informant	12th grade	37	–	Urban, Pakpatan
6	Informant	Illiterate	25	–	Rural, Pakpatan
1	GP	Private hospital	33	6 years	Urban, Sahiwal
2	HW	Public sector	31	10 years	Rural, Sahiwal
3	Nurse	Public sector	45	13 years	Rural, Okara
4	HW	Public sector	36	12 years	Rural, Okara
5	Midwife	Private sector	37	6 years	Urban, Pakpatan
6	GP	Public hospital	34	10 years	Rural, Pakpatan

GP = General Practitioner; HW = Health Worker

### Deliveries in private hospitals and prelacteal feedings

While deliveries in private health facilities are considered to be relatively safer than government facilities or home deliveries, our research indicated that prelacteals are more common in private health facilities. According to the interviews, the support staff of these facilities (such as traditional birth attendants, nurses, and paramedical staff) were seen to be supportive towards prelacteal feeding. This was best illustrated by a mother who shared her experience describing the unsolicited administration of a prelacteal by the Aaya (child attendant):

*“When my child was born at a private health facility, the Aaya gave the prelacteal to my child in the labor room without our consent and demanded money for her good gesture. She did not hand over the baby [to me] until she was paid.” (Informant, 3).*

Apart from the administration of prelacteals by hospital staff, parents also brought prelacteals into hospitals, or purchased them from medical stores located near the private health centers. Highlighting this, one physician in a private hospital made the following comment:

*“When women are admitted to the hospital for delivery, they also bring a bottle containing honey and rose water with their luggage to give prelacteal to the newborn after delivery. If someone forgets to bring the prelacteals, then they [caretakers] purchase the prelacteals from medical stores in the hospitals.” (Key-informant, 6).*

It is worth mentioning here that anyone can buy medicine and supplies from any medical store or hospital without a prescription in Pakistan, and several prelacteal brands (e.g., *Anmol Ghutti, Hamdard Ghutti, Asli Ghutti,* and *Janam Ghutti*) are available at medical stores. Moreover, there are no governmental or health facility policies that currently discourage the use or sale of prelacteals.

### Inadequate antenatal counseling

Antenatal visits represent key opportunities to counsel mothers on prelacteals and exclusive breastfeeding. However, such services are rarely accessible for mothers living in rural areas. Furthermore, mothers who are fortunate enough to receive antenatal care may not follow through with the advice given, choosing to instead follow traditional practices of prelacteal feeding. This was evident in the account of a physician who reported:

*“We tell the women to feed the colostrum immediately after birth instead of any prelacteal. But mothers do not follow our advice.” (Key-informant, 6).*

A mother corroborated this statement, stating:

*“Now the doctors condemn the prelacteal feeding, but prelacteal is our family tradition that must be carried out.” (Informant, 3).*

As these excerpts illustrate, prelacteal feedings are a well-established social norm. Therefore, future interventions will likely require comprehensive antenatal counseling about the potential adverse health complications of prelacteal feedings. Such counselling would not only be needed for expecting mothers, but also to other family members (specifically, mothers-in-laws).

### Myths about colostrum

Colostrum is the first form of milk produced by the mammary glands. It contains numerous antibodies that protect the newborn against disease. While most informants reported feeding colostrum to their newborn, some mothers from rural areas reported not giving colostrum to their neonates. They attributed this to various taboos that revolve around the belief that the colostrum is stale and harmful for the neonate’s health. Some rural mothers reported testing the colostrum before initiation of breastfeeding at home. One rural mother with four children explained the process she follows:

*“First, an ant is put into the colostrum to check the milk. If the ant dies, then we do not feed colostrum to baby and throw it away. If the insect does not die, then we feed it to the baby. If the insect dies, it means that the milk is poisonous. We also get it checked by local practitioners (Quacks); if they say the milk is not harmful then we feed it to the babies.” (Informant, 6).*

This excerpt signifies that some mothers in rural areas think the colostrum might be poisonous. The reasoning behind this belief stems from the idea that the colostrum remains in the mother’s breasts for several months, making it stale and dangerous to feed to children. Such beliefs drive some women to deprive their children of the colostrum until their transitional milk comes.

### Prelacteal as a family ritual

Our interviews also uncovered that families prefer the first prelacteal feed be given by a senior family member, or a virtuous individual. Families believe this practice will transfer the qualities and habits of the feeder into the child. A surprising finding from our study indicated that some families may even feed the saliva of the perceived virtuous individual as a prelacteal. This is illustrated in the account of a key informant presented below:

*“Parents prefer a virtuous person from the family to pour honey or saliva into the mouth of the neonate, so that child may acquire the personality traits and habits of that person in his/her future life. After feeding the prelacteal, mothers may start breastfeeding the child.” (Key-informant, 2).*

### Sunna of holy prophet

The Islamic ceremony of *Tahnik* consists of touching the lips of a newborn baby with a sweet substance, such as honey. During the lifetime of the Prophet, it is said that Muslims would bring their newborns to him so that he may perform *Taḥnīk* upon them. Consequently, the use of honey as a prelacteal has taken on a special meaning and is considered a sacred tradition of the prophet (Sunna). Owing to this sacred origin, the use of honey is imbued with cleansing properties, and many individuals now consider it to be a beneficial laxative that cleanses the baby’s stomach. This notion is illustrated in the excerpt below:

*“To feed the honey as prelacteal is the Sunna of our beloved Prophet. The Holy Prophet used to feed honey and dates to neonates immediately after birth. After delivery, I could have breastfed my child but my mother-in-law gave [the child] honey to clean his stomach and help to pass the meconium.” (Informant, 5).*

Therefore, the use of certain prelacteals have strong roots in religion and home medication. Honey is considered a sacred prelacteal, and parents prefer honey as the first feed for newborns to accomplish Sunna and to pass the meconium.

### Insufficient breast milk

Our qualitative interviews also revealed that mothers are rarely aware of the importance of early initiation of breastfeeding. Moreover, many mothers hold a strong belief that breast milk comes after the third day of delivery. Consequently, prelacteals are perceived to be the best option to satiate the child. Apart from mothers, this view is also propagated by community health workers, who suggest that mothers give prelacteals to supplement perceived insufficient breast milk:

*“Mother’s milk comes after three days even in case of normal delivery. A mother’s body does not produce milk after a couple of days of delivery; that is why mothers should give prelacteals to newborns” (Key-Informant, 5).*

Breast milk supply is tightly regulated by infant demand, and immediate breastfeeding is recommended to foster breast milk production. However, most of the mothers interviewed were not well aware of the best practices for successful breastfeeding. In certain cases, mothers often waited up to 3 days after delivery to start breastfeeding their newborns.

### Quantitative results

In the PDHS 2012–2013 survey, about half of the index infants were male, and the prevalence of prelacteal feeding did not differ by infant sex (see Table 2). The mean age of mothers was 26.94 (SD = 5.54) years, and more than half were illiterate (52%) and living in rural areas (54.6%). The mean age of children was 13.42 months (SD = 2.37). For prenatal care, a large proportion of mothers (60.6%) had less than four antenatal care visits, and nearly half (45.3%) of infants were born at home.

**Table 2:** Characteristic of dyads including in analysis of prelacteal feeding in Pakistan, PDHS 2012–13 (*n* = 1361)

Variables	All (***n*** = 1361) % (***n***)	Prelacteal feeding		***P*** - value
		Yes % (***n***)	No % (***n***)	
**Total Sample**	**–**	**64.7 (881)**	**35.3 (480)**	
**Maternal education**				
No education	52.5 (715)	63.3 (453)	36.7 (262)	< 0.001
Primary	15.4 (210)	75.1 (158)	24.9 (52)	
Middle	8.2 (112)	68.8 (77)	31.2 (35)	
Secondary	11.4 (155)	65.8 (102)	34.2 (53)	
Higher	12.6 (171)	53.2 (91)	46.8 (80)	
**Maternal age**				
15–24	33.1 (450)	66.5 (299)	33.5 (151)	0.27
25–34	52.8 (719)	64.9 (467)	35.1 (252)	
34 and above	14.1 (192)	59.9 (115)	40.1 (77)	
**Wealth index**				
Poorest	20.1 (274)	56.8 (156)	43.2 (118)	< 0.001
Poorer	20.4 (278)	61.2 (170)	38.8 (108)	
Middle	19.8 (269)	69.6 (187)	30.4 (82)	
Rich	19.6 (267)	74.5 (199)	25.5 (68)	
Richest	20.1 (274)	61.9 (170)	38.1 (104)	
**Sex of household head**				
Male	93.2 (1268)	64.0 (812)	36.0 (456)	0.030
Female	6.8 (93)	74.0 (69)	26.0 (24)	
**Place of residence**				
Urban	43.6 (593)	62.3 (370)	37.7 (223)	0.055
Rural	56.4 (768)	66.6 (511)	33.4 (257)	
**Region**				
Punjab	28.0 (381)	86.6 (330)	13.4 (51)	< 0.001
Sindh	22.1 (301)	56.5 (170)	43.5 (131)	
Khyber Pakhtunkhwa	20.2 (275)	73.1 (201)	26.9 (74)	
Baluchistan	15.0 (204)	62.3 (127)	37.7 (77)	
Islamabad	5.1 (69)	56.5 (39)	43.5 (30)	
Gilgit-Baltistan	9.6 (131)	10.7 (14)	89.3 (117)	
**Antenatal care**				
No	23.0 (313)	59.7 (187)	40.3 (126)	0.04
1–3 visits	37.6 (512)	68.4 (350)	31.6 (162)	
≥ 4 visits	39.4 (536)	64.1 (344)	35.9 (192)	
**Place of delivery**				
At home	45.3 (617)	67.2 (415)	32.8 (202)	< 0.001
Public health facility	19.5 (265)	51.9 (137)	48.1 (128)	
Private	35.2 (479)	68.7 (329)	31.3 (150)	
**Birth by cesarean section**				
No	86.2 (1173)	63.4 (743)	36.6 (430)	0.004
Yes	13.8 (188)	73.7 (138)	26.3 (50)	
**When child put to breast after delivery**				
Within one hour	23.0 (313)	38.7 (121)	61.3 (192)	< 0.001
2–24 h	49.4 (672)	59.2 (398)	40.8 (274)	
After one day	27.6 (376)	96.3 (362)	3.7 (14)	
**Sex of child**				
Male	50.7 (690)	63.6 (439)	36.4 (251)	0.21
Female	49.3 (671)	65.9 (442)	34.1 (229)	
**Birth order**				
First born child	23.7 (323)	70.5 (228)	29.5 (95)	0.008
Subsequent child	76.3 (1038)	62.9 (653)	37.1 (385)	

After delivery, nearly half of mothers (49.4%) reported delayed initiation of breastfeeding during the first day of life (2–24 h.), while a quarter of mothers (27.6%) reported that they initiated breastfeeding more than 24 h after delivery (Table 2). Almost two-thirds (64.7%) of respondents reported giving prelacteals to their children aged 0–23 months. The most common prelacteal was milk other than breast milk (24.5%), while over a fifth (21.8%) of mothers reported giving honey or sugar water (see Table 3).

**Table 3:** Analysis of preferred prelacteal feeding during the first three days of birth (*n*^a^ = 881; 64.7%)

Types of prelacteals	Frequency	Percentage
Milk other than breast milk	216	24.5
Honey/ Sugar water	192	21.8
Infant formula	119	13.5
Marketed Ghutti	132	15.0
Plain water	48	5.4
Fruit juice	44	5.0
Rosewater	41	4.6
Green tea	41	4.6
Ghee/ Butter or tea	28	3.2
Gripe water	20	2.3

^a^Only 881 dyads are included who gave a prelacteal feed during the first 3 days of birth

Binary logistic regression showed that maternal education, wealth index, region, antenatal care visits, place of delivery, cesarean delivery, timing of breastfeeding relative to delivery, and birth order were associated with prelacteal feeding (Table 4). Adjusted multiple logistic regression model (Table 4) with backward stepwise selection showed that maternal education, region, place of birth (hospital type or home birth), and timing of initiation of breastfeeding are associated with prelacteal feeding. Compared to highly educated mothers, mothers with primary education were two-times more likely to give prelacteals. When compared to Gilgit-Baltistan, living in the provinces of Punjab and Khyber Pakhtunkhwa was associated with substantially higher odds of prelacteal feedings. Additionally, it was found that children who were born at government hospitals were less likely to receive prelacteal feeds, compared to children born at private hospitals and home. Children who were breastfed immediately after birth were also much less likely to receive prelacteals, compared to children who breastfed after 24 h.

**Table 4:** Adjusted and unadjusted odd ratios (95% confidence interval [CI]) for factors associated with prelacteal feeding in Pakistan, (PDHS, 2012–2013)

	Likelihood of prelacteal feeding					
Variables	OR^**a**^	***P*** - value	95% CI	AOR	***P*** - value	95% CI
**Education of mother**						
No education	1.53	0.013	1.09, 2.15	1.28	0.369	0.78, 1.91
Primary	2.65	< 0.001	1.71, 4.09	2.28	0.002	1.35, 3.85
Middle	1.93	0.010	1.93, 1.17	1.88	0.041	1.02, 3.47
Secondary	1.69	0.021	1.08, 2.64	1.80	0.034	1.04, 3.09
Higher	1			1		
**Wealth index**						
Poorest	0.80	0.223	0.57, 1.13			
Poorer	0.97	0.856	0.68, 1.36			
Middle	1.41	0.058	0.98, 2.01			
Rich	1.80	0.002	1.24, 2.60			
Richest	1					
**Sex of household**						
Male	0.62	0.50	0.38, 0.99			
Female	1					
**Region**						
Punjab	54.07	< 0.001	28.8, 101.3	24.92	< 0.001	12.6, 49.1
Sindh	10.84	< 0.001	5.95, 19.74	7.03	< 0.001	3.65, 13.5
Khyber Pakhtunkhwa	22.70	< 0.001	12.31, 42.0	16.21	< 0.001	8.30, 31.6
Baluchistan	13.78	< 0.001	7.39, 25.68	12.20	< 0.001	6.14, 24.3
Islamabad	10.86	< 0.001	5.23, 52.55	9.60	< 0.001	4.31, 21.4
Gilgit-Baltistan	1			1		
**Antenatal care**						
None	0.83	0.040	0.62, 1.10			
1–3 visits	1.20	0.250	0.93, 1.56			
≥ 4 visits	1					
**Place of delivery**						
At home	0.93	0.604	0.72, 1.20	0.997	0.89	0.69, 1.37
Public health facility	0.49	< 0.001	0.36, 0.66	0.462	0.02	0.43, 0.95
Private health facility	1					
**Birth by Cesarean section**						
No	0.62	0.007	0.43, 0.87			
Yes	1					
**When child put to breast**						
Within one hour	0.02	< 0.001	0.14, 0.44	0.03	< 0.001	0.01, 0.61
2–24 h	0.05	< 0.001	0.32, 0.98	0.07	< 0.001	0.43, 0.95
After one day	1			1		
**Birth order**						
First born child	1.40	0.013	1.07, 1.84			
Subsequent child	1					

^a^Binary logistic regression

Education of mothers, region, place of delivery, and when child put to breast

### Triangulation of findings

**Fig. 1:**
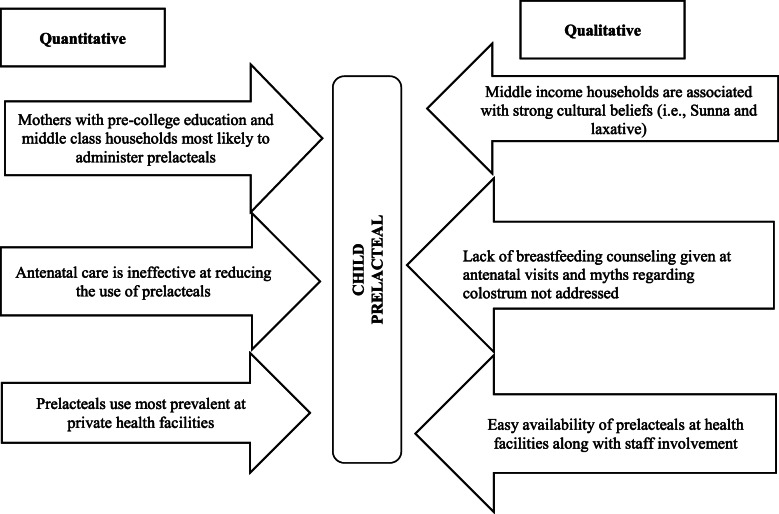
Triangulation of the findings

As a result of the mixed-methods design, we were able to triangulate findings from our qualitative analysis with the quantitative assessment (see Fig. 1). The figure summarizes the nexus between qualitative and quantitative findings, and provides a holistic perspective to our findings.

## Discussion

Prelacteal feeding is mostly practiced in LMICs, owing to cultural traditions and its perceived health benefits. This is the first mixed-methods study seeking to understand the sociodemographic and cultural factors influencing prelacteal feeding in Pakistan. Our quantitative analyses, using PDHS (2012–13) data, revealed that a majority of children (64.7%) under 2 years of age received prelacteal feeds in Pakistan. This finding is critical given that prelacteal feeding has been shown to delay the early initiation of breastfeeding, a practice that is detrimental to neonatal health. Additionally, our findings suggest mothers, irrespective of age and place of residence, gave prelacteals to both male and female neonates. Moreover, there was also no difference in prelacteal feeding between highly educated and illiterate mothers. However, mothers who had pre-college education (i.e., 5th to 12th grade) were more likely to give prelacteals compared to both illiterate mothers and mothers with higher education. This finding has also been depicted in studies from Nigeria [35] and Ethiopia [34, 36–38]. A similar pattern was also observed in relation to household income. Our analyses indicated that households of both low and high income were slightly less likely to give the prelacteals, compared to middle or upper-middle income households. This finding was surprising, particularly since several studies from LMICs found no association between household income and prelacteal feeding, specifically in Nepal [39, 40], Nigeria [35], and Egypt [4].

This indicates that prelacteals have a distinct importance in different socio-economic classes in LMICs, and caretakers use prelacteals according to their sociocultural preferences. Within the context of Pakistan, it is plausible that the most disadvantaged households may be too poor or burdened to purchase prelacteals, while more affluent households may be more influenced by Western medical discourse at the expense of traditional practices. However, middle-income households are viewed as more value-oriented and inclined to preserve cultural traditions. Our interviews revealed several such traditions. For example, value-oriented families preferred that a virtuous individual or family member administer prelacteals to transfer the positive characteristics of the feeder to the newborn. As another example, owing to its importance as a practice of the prophet (Sunna), middle-income families also preferred the use of honey as a prelacteal. This is consistent with studies from Muslim countries that found the use of prelacteals is related to the preservation of religious beliefs [41–45].

Antenatal care visits provide health care professionals opportunities to counsel pregnant mothers on optimal breastfeeding practices and newborn care. Therefore, we were surprised when our quantitative analyses revealed that mothers who did not receive any antenatal care were less likely to give prelacteal feeds. It is likely that these results can be explained by taking into consideration that low income households are unable to visit antenatal clinics due to accessibility and financial constraints. Consequently, a larger pool of antenatal visitors may consist of women from middle income households, a segment of the population most likely to implement prelacteals. Results from the national survey corroborate this account, as they indicate that the poorest households in Pakistan immediately start breastfeeding and are less likely to implement prelacteal feeds [46].

Despite the survey results, our qualitative interviews revealed that several participants reported not receiving effective breastfeeding counseling during antenatal visits. Further, several studies from Ethiopia have documented lack of infant feeding counseling during antenatal care as a major determinant of prelacteal feeding [33, 34, 47, 48]. Effective counseling on infant feeding practices can serve as an avenue to address two findings identified as potential predictors of prelacteal feeding by our study: 1) parity, and 2) myths about colostrum. Primiparous women were more likely to administer prelacteals. This practice may reflect upon a general tendency for mothers to be more sensitive about neonatal care practices when expecting their first child, and that primiparous women are more likely to rely on support from others when establishing breastfeeding.

Concerning parity, and given that our study revealed that some prelacteals are believed to be imbued with strong religious meanings and are associated with health benefits, it is likely that these first-time mothers are more open to administering them due to these beliefs. Incorporating breastfeeding education into antenatal care can inform mothers on the importance of the early initiation of breastfeeding, a fact that is neglected when mothers opt to administer prelacteals. Secondly, our interviews revealed that some mothers held the view that colostrum may be harmful to neonates and should be discarded. The practice of discarding colostrum has been observed in other settings due to a perceived fear of child’s abdominal pain [49], beliefs that colostrum is old or stale [48–50], and beliefs that colostrum is associated with mortality [50]. We also learned that some mothers and healthcare providers believed that the breast milk transition from colostrum to milk takes 3 days after delivery. In light of this, different prelacteals are given to satiate the child during this perceived transitioning phase. In many cases, mothers may deliberately delay breastfeeding until the implementation of the prelacteal feed. This insight is noteworthy given the strong association between prelacteal feeding and the delayed initiation of breastfeeding. It is essential that such misconceptions be addressed through antenatal care.

Finally, we explored the role of healthcare facility type, including private health facilities, in facilitating prelacteal feeding. It is interesting to see that there was no difference in prelacteal administration to newborns who were delivered at home and in private health facilities. However, children born at public health facilities were less likely to receive prelacteals. Our interviews revealed that in some cases, attendants in private health facilities administer prelacteals to neonates without soliciting consent from the parents. Taken together, the prevalence of prelacteal feeding in Pakistan may be highest due to this practice by caretakers and hospital staff. However, previous reports have highlighted that home deliveries were associated with prelacteal feeding, but other studies from Nepal [39], Ethiopia [47] and Pakistan [51] did not observe an association between prelacteal feeding and place of delivery. The difference in our findings may be explained by taking into consideration that impoverished mothers in Pakistan may be unable to afford both a private hospital delivery and prelacteal feeds. This may make them more likely to deliver their babies at home or in public facilities, and breastfeed immediately.

### Strength and limitations

This mixed-methods research is a pioneering approach to understand sociodemographic factors associated with prelacteal feeding in Pakistan. This approach represents a strength of this research, as the findings reported have been triangulated from different sources. Our qualitative study provided further insight into the quantitative findings to better understand the widely accepted practice of prelacteal feedings. However, our quantitative findings are from a cross-sectional survey, and limit our ability to infer causality. Additionally, the qualitative interviews were conducted in only three districts from Punjab and our findings may not be generalizable to other areas or regions of Pakistan.

## Conclusions

Pakistan has the highest global rate of neonatal mortality and child malnutrition due to substandard infant feeding practices and non-exclusive breastfeeding. Prelacteal feeding is customarily practiced and is a socially normative behavior in Pakistan. Many studies have reported associations between prelacteal feeding and child morbidity and mortality across the world. In our mixed-methods study, we found that mothers with pre-college education, primiparous women, mothers delivering at private health facilities, and mothers delaying the early initiation of breastfeeding were more likely to administer prelacteals to their infants. Apart from the socio-economic and demographic factors, we also identified several cultural practices that propagated the practice of prelacteal feeding.

This study provides necessary insight for policies, programs, and individuals promoting early initiation of breastfeeding and exclusive breastfeeding. Given the importance of early initiation of breastfeeding, along with the role of prelacteals in delaying this practice, it is essential that policies and educational programs be designed in ways that incorporate these findings to achieve the desired behavior change (i.e., early initiation of breastfeeding) and improve neonatal health outcomes. Without addressing these identified sociocultural influences and psychosocial barriers that impact breastfeeding initiation and avoidance of prelacteal feeding, interventions may not be successful in their efforts and families will continue to administer prelacteals to their children, leading to increased neonatal morbidity and mortality.

## Data Availability

The DHS dataset is publicly available in the DHS repository, https://www.dhsprogram.com/data/available-datasets.cfm. The qualitative data generated during the current study are not publicly available, but a restricted use dataset is available from the corresponding author on reasonable request.

## Abbreviations

AOR: Adjusted Odds Ratio
PDHS: Pakistan Demographic and Health Survey
SD: Standard Deviation
LMIC: Low and Middle Income Countries
WHO: World Health Organization
